# Beta-glucans induce cellular immune training and changes in intestinal morphology in poultry

**DOI:** 10.3389/fvets.2022.1092812

**Published:** 2023-01-09

**Authors:** Hadar Bar-Dagan, Ofer Gover, Natalie Avital Cohen, Vaclav Vetvicka, Israel Rozenboim, Betty Schwartz

**Affiliations:** ^1^Institute of Biochemistry, Food Science and Nutrition, Robert H. Smith, Faculty of Agriculture, Food and Environment, Hebrew University of Jerusalem, Rehovot, Israel; ^2^Department of Animal Sciences, Robert H. Smith, Faculty of Agriculture, Food and Environment, Hebrew University of Jerusalem, Rehovot, Israel; ^3^School of Medicine, Department of Pathology, University of Louisville, Louisville, KY, United States

**Keywords:** beta-glucans, HD-11 cells, immune training, biological response modifiers, goblet cells

## Abstract

**Introduction:**

Beta-glucans are known as biological response modifiers due to their ability to activate the immune system. This research aimed to determine the efficacy and safety of feeding beta-glucans from various sources on the immune status and intestinal morphology of chickens.

**Methods:**

To this end we used *in vitro* and *in vivo* set-ups. In the *in vitro* set-up the chicken macrophage cell line HD-11 was used to measure the response of the chicken immune cells to beta-glucans extracted from algae and mushrooms on immune-related gene expression and associated activities. Additionally, we conducted two *in vivo* experiments using either beta-glucans extracted from yeast or mix of yeast and mushrooms beta-glucans as part of the chicks feed in order to test their effects on the chick intestinal morphology.

**Results:**

In the *in vitro* set-up exposure of HD-11 cells to a concentration of 1 mg/ml of algae and mushroom beta-glucans resulted in significantly higher expression of 6 genes (TNFα, IL4, IL6, IL8, IL10, and iNOS_2_) compared to control. The release of nitrite oxide (NO) to the medium after exposure of HD-11 cells to mushrooms or algae beta-glucans was significantly increased compared to control. Additionally, significantly increased phagocytosis activity was found after exposure of the cells to algae and mushroom beta-glucans. In the *in vivo* set-up we observed that the length of the villi and the number of goblet cells in the ileum and the jejunum in the beta-glucan fed chicks were significantly augmented compared to control, when the chicks were fed with either yeast or yeast and mushroom beta-glucans mix.

**Discussion:**

In conclusion, dietary supplementation of poultry with beta-glucan exerts significant and positive effects on immune activity and the intestinal morphology in poultry.

## 1. Introduction

Bacterial infections in poultry represent an essential issue of farming that affects animal welfare, health, and productivity. Infections due to bacteria in poultry are an important concern for the bird's health and efficient production. So far, farmers have used antibiotics to control diseases involving bacterial infections in poultry. Chickens have also been fed with low concentrations of antibiotics to improve daily weight gain and feed efficiency through alterations in disease suppression (1). Due to the massive use of antibiotics, a surge in the development and spread of antibiotic-resistant bacteria has become a major cause for concern. Antimicrobial resistance is a natural event that takes place when bacteria no longer respond to antibiotics to which they were previously susceptible and that were previously active in treating infections caused by these microorganisms (2). Over the past few decades, no major new types of antibiotics have been produced. Almost all known antibiotics are increasingly losing their effectiveness against pathogenic microorganisms, especially those used in poultry farming (3). As the incidence of antibiotic resistance has become a serious problem, there is increased pressure on producers to reduce the use of antibiotics in the poultry farming industry (2).

With the reduced availability of antibiotics, poultry producers work to reduce the use of antibiotics and look for feed additives to stimulate the immune system of chickens to resist microbial infection (4). Moreover, there has been increased interest in “natural” feed additives that can stimulate the immune system of poultry. In addition, a significant push to produce antibiotic-free poultry is gaining popularity among the general public (5).

Several studies show that beta-glucans may play a role in replacing antibiotics and stimulate the immune system (6). Glucans are carbohydrates made of complex glucose polymers that provide the primary structure found in the cell wall of yeast, fungi, algae, and cereal grains such as oats and barley (7). The structures of glucans vary depending on their source and the type of linkages on the glucose polymers (8). The nature of these linkages will affect the functionality of the molecules.

Beta-glucans are transported to the small intestine, then pass through the Peyer's patches in the gut-associated lymphoid tissue (GALT), and subsequently, they are transported around the body (9). The effect of beta-glucans is mediated by its binding to specific receptors. It stimulates macrophages, secretion of antibodies, and increases the activity of natural killer cells. Similar to mammals, avian macrophages synthesize cytokines and chemokines such as Tumor Necrosis Factor-alpha (TNFα), Interleukin 4 (IL4), Interleukin 6 (IL6), Interleukin 8 (IL8), Interleukin 10 (IL10), and interferon gamma (IFN-γ) (10, 11). In addition to the direct stimulation of specific and non-specific immunity, glucans can also influence the expression of immune-related genes and proteins. The stimulation in immune functioning serves to combat the adverse effects of enteric infection or immune suppression due to high-stress rearing conditions (9).

Beta-glucans were reported to be effective in promoting the growth of broiler chickens and improving their meat quality (12). Beta-glucans have been shown to improve gut health in poultry subjected to a bacterial challenge and to increase the flow of new immunocytes into the various lymphoid organs (13). Beta-glucans can increase macrophage functionality (14), affect intestinal morphology, and function as anti-inflammatory immunomodulators (7). Dietary Mannan-Oligosaccharide (MOS) Yeast Extract containing MOS and beta-glucans supplementation to turkey poults can enhance the number of goblet cells and upregulate mucin-2 production from goblet cells (15), improves villus height and crypt depth and their ratio in the duodenum and ileum (16). Moreover, consumers can more easily accept feed enriched with beta-glucans with more confidentiality than antibiotics-treated poultry (5). We propose that when using a declaration “glucans introduced into the poultry feed” will be always associated with “antibiotic free poultry,” and this information can be easily transferred to the consciousness of the consumer. Beta-glucan mixtures may be implemented to obtain optimal anti-inflammatory, immunomodulatory, growth performance and optimized intestinal morphology and histology responses in poultry (17, 18).

The specific activity of each beta-glucan subtype is unknown, and no direct comparison of beta-glucans derived from different sources has been made in poultry. Despite numerous glucans isolated from various sources, the decision about the most active glucans in avian or any other animal model has not been reached, sometimes leading to contradictory results. Because there is extensive variation in beta-glucan structure based on their origin, their effectiveness in modulating the immune system can vary (8). Nonetheless, it is generally accepted that 1,3 and 1,6-beta-glucans, derived from yeast and mushroom, are considered the most effective form available due to their complex network of carbohydrate branching (19, 20). Therefore, we focused on using beta-glucans, originating from yeast, mushroom, and algae, as putative immunomodulators in poultry. Our goal was to find the optimal beta-glucan type and concentration to exert the optimal immunological training and adaptations on intestinal morphology activities in poultry using *in vitro* and *in vivo* setups. The results of our study may contribute to the general goal of reducing or even eliminating antibiotics which may be exceptionally beneficial to farmers to prevent economic losses and to consumers of chicken that seek antibiotic-free meat (21). The results may contribute commercially necessary knowledge on this critically not adequately explored agricultural field.

## 2. Materials and methods

### 2.1. Beta-glucans

(1) An algae glucan extract, used in *in-vitro* assays, was kindly provided by Quegen Biotech (S. Korea). (2) Yeast (Saccharomyces cerevisiae) glucans (Glucan #300) used in *in-vivo* assays were kindly provided by Transfer Point (Columbia, SC, USA). Algae beta-glucans were used for the *in-vitro* assays since these beta glucans are easily soluble in water. Yeast (Saccharomyces cerevisiae) glucans are completely insoluble in water or any other solvents thus we could not use them in the *in vitro* assays. Moreover, the algae beta glucans cost is extremely high thus they are not feasible for using for feeding purposes, thus we elected for the feeding experiments to use yeast glucans (the ratio of alpha to beta glucans in both preparations is similar) and algae beta glucans for the *in vitro* assays. For *in-vitro* assays glucans were dissolved in double distilled water (DDW) to a final concentration of 50 mg/ml of the extract in a water bath for 1 h at 80°C. The solution was sterilized in an autoclave by heating at 121°C for 30 min. The solution was then diluted in Dulbecco's Modified Eagle Medium (DMEM) to a final concentration of 5, 1, 0.5, and 0.05 mg/ml. (3) Mushroom glucans were extracted from King oyster mushrooms (kindly provided by Tekoa farms, Israel). Mushrooms were ground to fine powder in dry ice. Mushroom powder was dissolved in distilled water, in a concentration of 1 g of mushrooms powder in 10 ml and autoclaved at 121°C for 30 min. The solution was then pelleted at a speed of 13,000×*g* at 10°C for 15 min; the liquid phase was transferred to ethanol to a final concentration of 1:2. The solution was then stored at a temperature of −20°C overnight. After about 24 h, a float is formed. The float was dried in a fume hood for 24 h. The dry float was frozen at −80°C overnight and lyophilized to obtain a uniform powder (22). For *in-vitro* experiments the mushrooms extracted glucans were dissolved in the solvent dimethyl sulfoxide (DMSO) to a final concentration of 5 mg/ml of the beta glucan extract in a water bath for 1 h at 90°C and diluted in DMEM at the final concentration of 5, 1, 0.5, and 0.05 mg/ml. *α* and *β* glucans were quantified using Megazyme beta-glucan assay kit (Megazyme, Ireland) according to manufacturer protocol.

### 2.2. Cell culture

The chicken HD11 macrophage cells were kindly provided by Dr. Elisabeth Kowenz-Leutz (Max Delbrueck Center, Berlin, Germany) and grown in 75 mm^2^ flasks with 18 ml DMEM growth medium supplemented with 8% fetal bovine serum (FBS), 2% chicken serum, 1% penicillin, and streptomycin. Then the cells were incubated at 37°C and 5% CO_2_ until they attained 95% confluency. For gene expression and Nitric Oxide analysis cells were grown in 6-well plates (1 × 10^6^ cells/ml). After 48 h, the medium was removed, and fresh DMEM medium with Lipopolysaccharide (LPS) (from *E. Coli*, O111:B4; Sigma L4130 (used at specified concentrations as indicated below) or glucans were added and used as a pro-inflammatory stimulant. Cells were harvested for RNA and supernatants were collected and stored at −80°C.

#### 2.2.1. Gene expression

RNA was extracted from HD11 cells using NucleoSpin RNA, Mini kit (MN, Germany), according to manufacturer instructions. RNA was quantified using Nanodrop 2000 (ThermoFisher USA). 2 μg of RNA was used to synthesize cDNA using qScript cDNA Synthesis Kit (Quanta bio, USA).

Real-time qPCR was performed using a fast SYBR green master mix (ABI, USA) on Quant studio 1 machine (ABI, USA). For normalization of gene expression in all reactions, we used the GAPDH gene. Primers for relative gene expression are presented in Table 1. Normalization and quantification were performed using either the std curve of serial 1:5 dilutions or the ddCt method (23).

**Table 1:** List of primers tested for relative gene expression.

**Gene**	**Primer F**	**Primer R**
GAPDH	CCTAGGATACACAGAGGACCAGGTT	GGTGGAGGAATGGCTGTCA
TNFa	CGCTCAGAACGACGTCAA	GTCGTCCACACCAACGAG
IL-4	GCTCTCAGTGCCGCTGATG	GAAACCTCTCCCTGGATGTCAT
IL-6	CAAGGTGACGGAGGAGGAC	TGGCGAGGAGGGATTTCT
IL-8	TCCTGGTTTCAGCTGCTCTG	TGGCGTCAGCTTCACATCTT
IL-10	ACAAAGCCATGGGGGAGTTC	GTTAAGCTGCCATTGAGCCG
IFN-γ	AGCTGACGGTGGACCTATTATT	GGCTTTGCGCTGGATTC
Cytochrome C	ACGCAAAACAGGACAAGCTG	TCAGAGTATCCTCACCCCAAGT
BAX	TCCTCATCGCCATGCTCAT	CCTTGGTCTGGAAGCAGAAGA
BCL-2	GATGACCGAGTACCTGAACC	CAGGAGAAATCGAACAAAGGC
Caspase-9	CGAAGGAGCAAGCACGACAG	CCGCAGCCCTCATCTAGCAT
iNOS	AACTCTCACAAAAACACGAAGCA	TTGTGTGATGTGGGAACGCT

#### 2.2.2. Nitric oxide colorimetric assay

Nitrate/Nitrite colorimetric assay kit (Cayman Chemical, USA) was used to quantify the amount of released nitric oxide to the medium. The process was done according to the manufacturer's protocol. Briefly, 200 μL of Assay Buffer was added to empty 96-wells, followed by 80 μL of the sample medium. 10 μL of Enzyme Cofactor was added to each well, followed by 10 μL of Nitrate Reductase Mixture for 1 h at room temperature. The wells were supplemented with Griess Reagent for 10 min of incubation and transferred to a plate reader to read the absorbance at 540 nm (BioTek synergy H1, Agilent technologies CA, USA).

#### 2.2.3. Phagocytosis assay

The macrophage phagocytosis activity was assessed using the kit pHrodo™ Red Zymosan BioParticles (“Zymosan,” Life Technologies, USA) according to the manufacturer's protocol. Briefly, HD11 cells were grown in 96-well plates (1 × 10^6^ cells/ml) for all experiments. After 48 h, the medium was removed, and a fresh DMEM medium with either algae beta-glucans (1 mg/ml), mushroom beta-glucans (1 mg/ml), or LPS (100 ng/ml, prepared in sterilized DDW) was added and used as a stimulant activator for 1 h. The BioParticles provided by the kit were vortexed and resuspended homogeneously in clear DMEM (pH = 7.4) and then sonicated for 10 min (Bioruptor; Diagenode, Denville, NJ) in order to homogeneously disperse the particles. The medium was then removed from the cultures, replaced with the dispersed “Zymosan particles” 100 μL/well, and incubated at 37°C with 5% CO_2_ for another 1–1.5 h. Phagocytosis activity in the attached cells was measured using a plate reader using excitation of 560 nm and emission of 590 nm (BioTek synergy H1, Agilent technologies CA, USA).

### 2.3. *In vivo* experiments

All experimental procedures were carried out under the approval of the ethics committee of the Hebrew University of Jerusalem. Two experiments were conducted at the poultry unit and research laboratories at the Faculty of Agriculture, Food and Environmental Sciences, Israel. All animal experiments were done under the care and supervision of Prof. Israel Rozenboim's. One of the *in vivo* experiments tested the effects of treatment with beta-glucans extracted from yeast at various concentrations; on the growth and development of broilers. The second *in vivo* experiment tested the effects of treatment with two types of beta-glucans isolated from yeast and mushrooms, at various concentrations. The experiments tested the growth and development of broilers and their gut health. Briefly, 1-day-old Fertile Ross 308 broilers were randomly allotted by sex (male and female) into a complete randomized block design experiment in a 3 × 2 factorial design for a 5-week feeding trial. Broiler chickens were subjected to a standard commercial diet supplemented with different levels of beta-glucan: No beta-glucans (control), Beta-glucans at a ratio of 250 mg/kg, and 1 g/kg feed were mixed with a standard commercial feed (basic feed). The first *in vivo* experiment was on February 2021 at the Faculty of Agriculture experimental hen house in Rehovot Israel. 150 chickens were divided according to gender (75 male and 75 females) and subdivided to three treatment groups. Control, 250 mg/kg feed beta glucans (Glucan #300, 89% beta-glucans), 1 g/kg feed glucans. The second *in vivo* experiment was conducted on April 2022. 60 chickens were divided according to gender (30 male and 30 females) and subdivided to three treatment groups. (1) Control (2) 250 mg/kg beta-glucans from yeast and cultivated mushrooms, in a 3:1 yeast-to-mushroom ratio into the feed; (3) 1 g/kg beta-glucans from yeast and cultivated mushroom in a 3:1 yeast-to-mushroom ratio into the feed. This feeding regime was elected based on preliminary results in a small number of birds (not shown) demonstrating positive effects on parameters measured for yeast beta-glucans. At the age of 35 days birds were sacrificed and paraffin slides were created as described below. During the whole period of the experiment, all birds had free access to feed and water. Birds were raised on two littered floor rearing rooms partitioned into 15 pens, each at a density of 10 birds per pen for each sex. The room temperature was centrally regulated and ventilated. All broilers were exposed to a 23 L:1D photoperiod. At 35 days of age, birds were sacrificed by CO_2_ asphyxiation, and an autopsy was conducted. Segments of the small intestine were obtained and fixed in a 4% buffered formalin solution. Serial 4 μm transverse sections from the ileum and jejunum were cut and stained for morphological evaluation (H&E stain). The villi length and the number of goblet cells were measured and counted using ImageJ software (National Institutes of Health, Bethesda, version 1.53s).

#### 2.3.1. Alcian blue-periodic acid: Schiff (AB-PAS) staining

AB-PAS staining of paraffin-embedded sections and formalin-fixed slides was performed according to the manufacturer's instructions [Periodic Acid-Schiff (PAS) Stain Kit (Scytek Laboratories)]. Briefly, 3% acetic acid solution was added dropwise onto tissue sections, followed by 5–10 drops of Alcian blue solution (pH 2.5). The slides were stained for 25–30 min and then added 5–10 drops of the periodic acid solution for 5 min. Slides were rinsed and added with Schiff's solution for 20 min, followed by Hematoxylin staining. Sections were mounted and visualized under the microscope and goblet cells were counted manually.

## 3. Statistics

All statistics were performed on JMP pro 14 (SAS Institute Inc., Cary, Nc, 1989-2019); data are expressed as mean ± SE. Comparison between means of more than two groups was analyzed using means ANOVA and Tukey HSD.

## 4. Results

### 4.1. Incubation of HD11 macrophages with algae β-glucans

Out of 11 genes assayed 6 showed differential expression in response to exposure to LPS. Expression was tested at different time points. TNFα, IL4, IL6, INFγ, Cytochrome C and BAX exibited maximal expression after 4 h of incubation with 100 ng/ml LPS (Supplementary Figure 1). IL8 showed maximal expression at 8 h with 100 ng/ml LPS (Supplementary Figure 1D), anti-inflamatory cytokine IL10 peaked after 2 h with 200 ng/ml LPS (Supplementary Figure 2A), BCL-2 peaked after 24 with 200 ng/ml LPS and Caspase 9 after 4 h with 200 ng/ml as well (Supplementary Figures 3A, B). iNOS_2_ significatly increased after 8 h and 50 ng/ml LPS (Supplementary Figure 3C).

**Figure 1:**
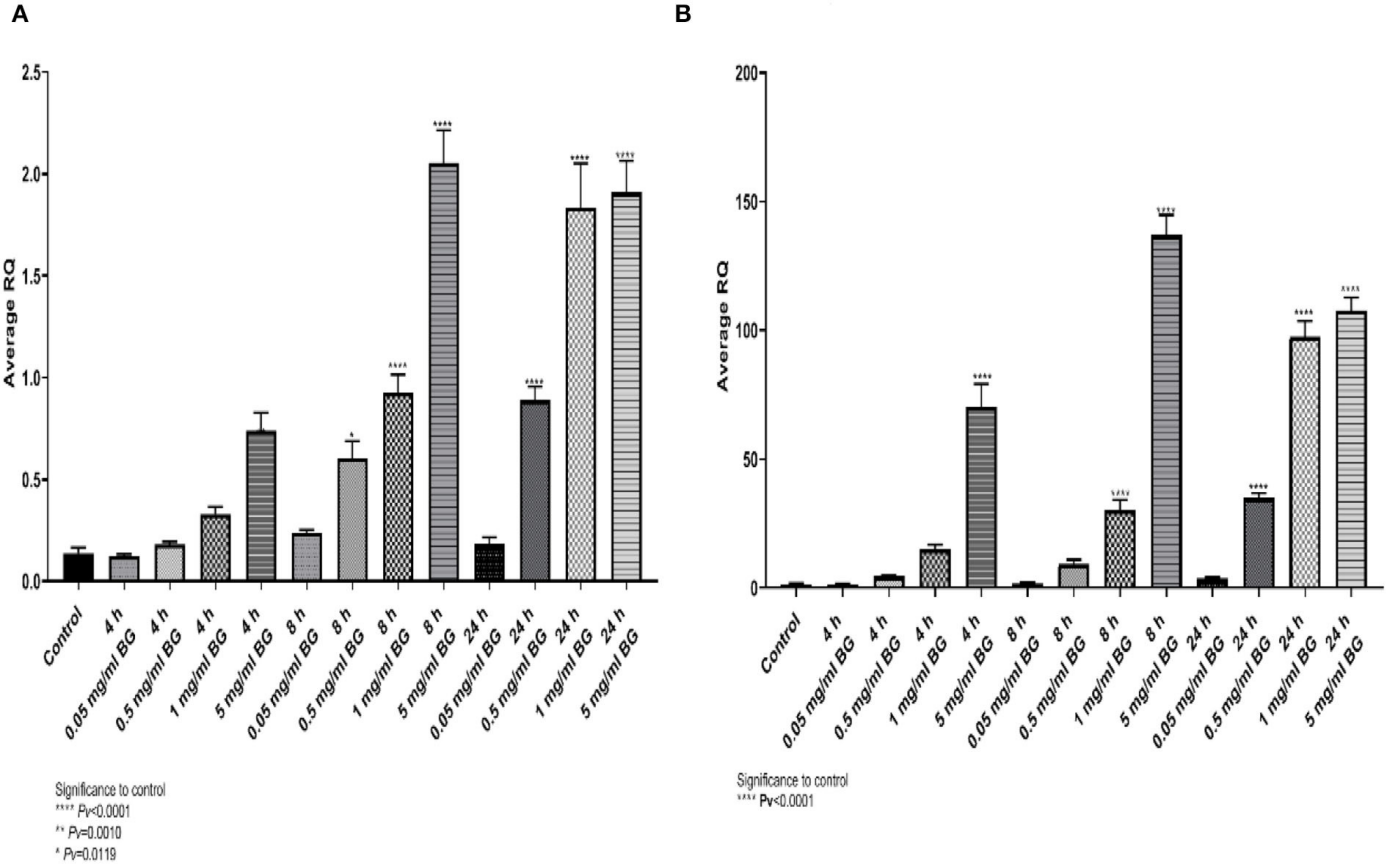
**(A)** Average relative expression for the gene TNFα, **(B)** the relative fold gene expression (DDCT) for the gene IL4, after exposure to algae beta-glucans at 3-time points and 4 concentrations (n = 6). ****Pv < 0.000.1, **Pv = 0.0010, *Pv = 0.0119—compared to control.

Algae beta-glucans stimulated dose-dependently gene expression of TNFα and IL4. The maximal expression obtained for TNF-α (Figure 1A) was detected at 5 mg/ml after 8 h incubation and 1 and 5 mg/ml for 24 h incubation. For IL4, the highest expression was reached at 5 mg/ml for 8 h followed by 1 and 5 mg/ml for 24 h (Figure 1B).

**Figure 2:**
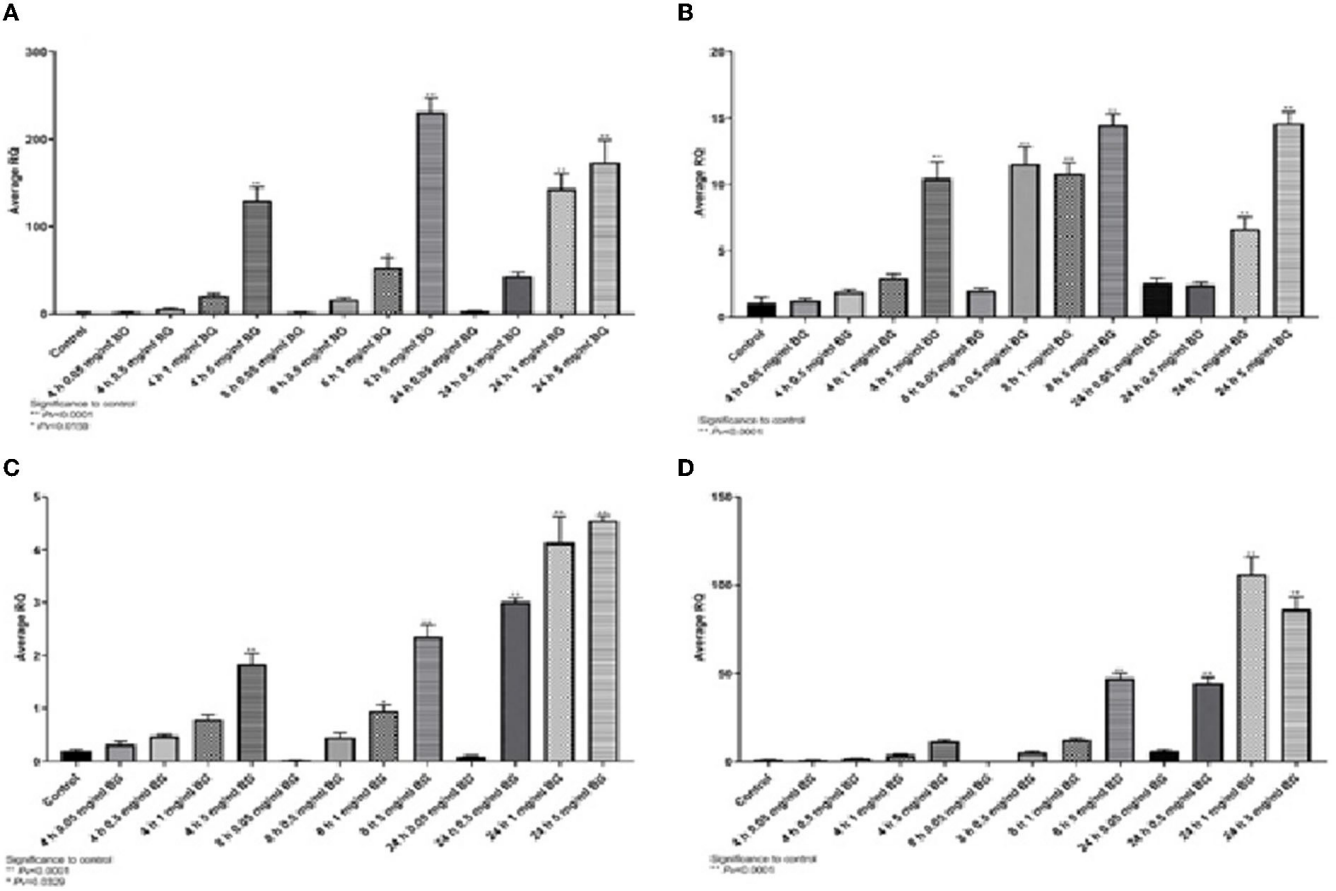
Expression of cytokine genes at different algae β-glucan concentrations and incubation times. **(A)** IL6, **(B)** IL10, **(C)** IL8 and **(D)** iNOS_2_.

A similar expression pattern was shown for IL6 and IL10 (Figures 2A, B), where the expression peaked at 8 h and 5 mg/ml whereas for IL8 and iNOS_2_ the highest levels were detected at 24 h (Figures 2C, D). The results indicate upregulation of cytokine gene expression resulting from incubation with algae beta-glucans.

#### 4.1.1. Release of nitric oxide

**Figure 3:**
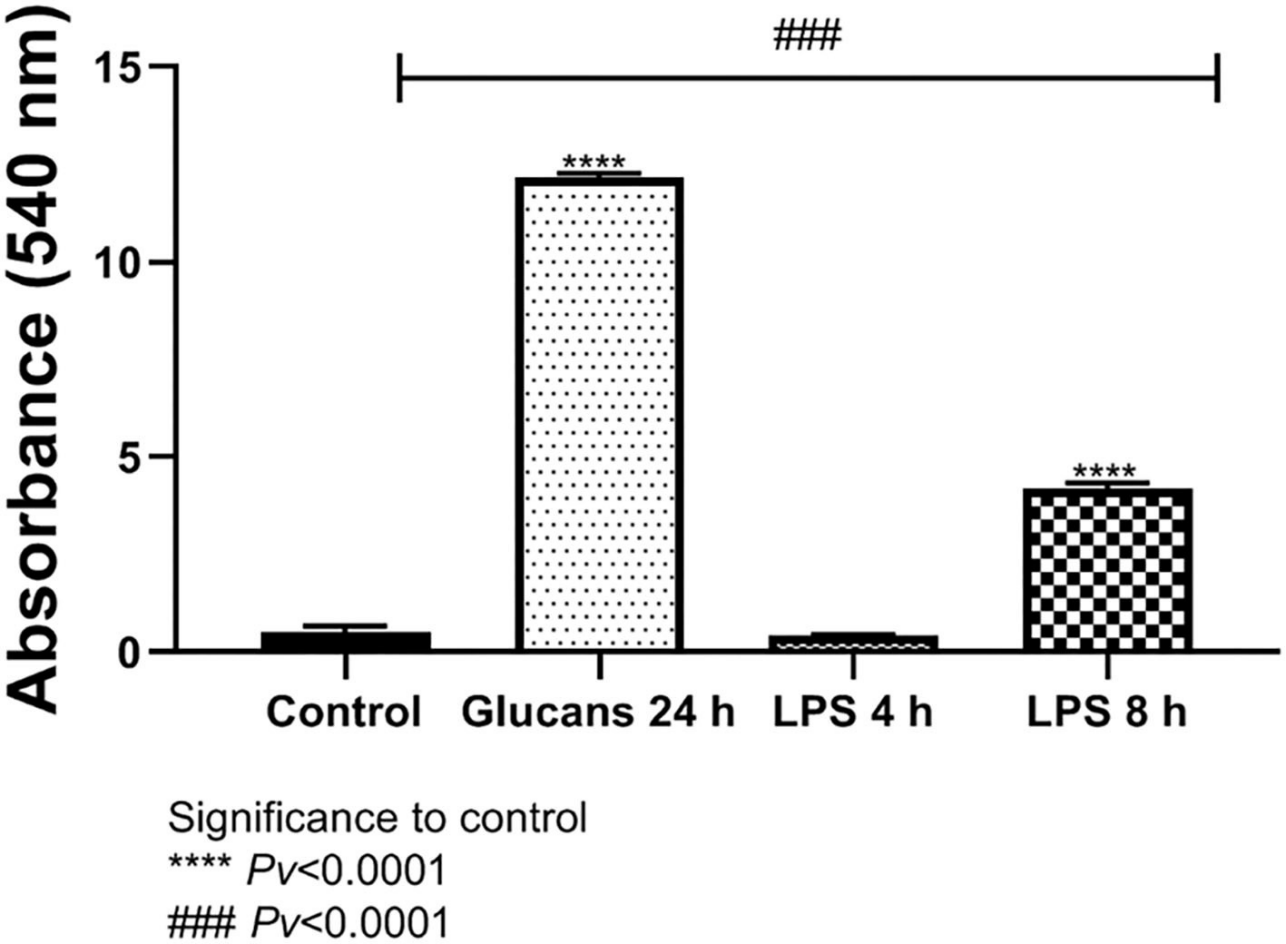
Release of NO to the medium after exposure to algae beta-glucans glucans for 24 h, or LPS. ****Pv < 0.0001 compared to the control group. ^###^Pv < 0.0001 compared the exposure to beta-glucans in contrast to LPS for 4 and 8 h (n = 5).

Nitric oxide is is an important molecule related to the antimicrobial effects of macrophages and is a chemical indicator of inflammatory response. The release of NO was significantly different from the control levels after exposure to 1 mg/ml of algae beta-glucans for 24 h. The release of NO after stimulus with LPS at a concentration of 100 ng/ml for 4 h of stimulation showed no significant difference from the control; however, after 8 h was significantly different from control. The release of NO to the medium after exposure to algae beta-glucans at a concentration of 1 mg/ml for 24 h was significantly higher than even after the exposure to LPS for 4 h as well as for 8 h (Figure 3).

### 4.2. Comparison of beta-glucans extracted from algea and mushrooms

Analysis of glucan content revealed that algae extracts consisted of 59.4% beta-glucans whereas mushroom extracts contain 8.5% beta-glucans. HD11 macrophages were incubated with 1 mg/mL of each extract for 24 h.

#### 4.2.1. Gene expression

**Figure 4:**
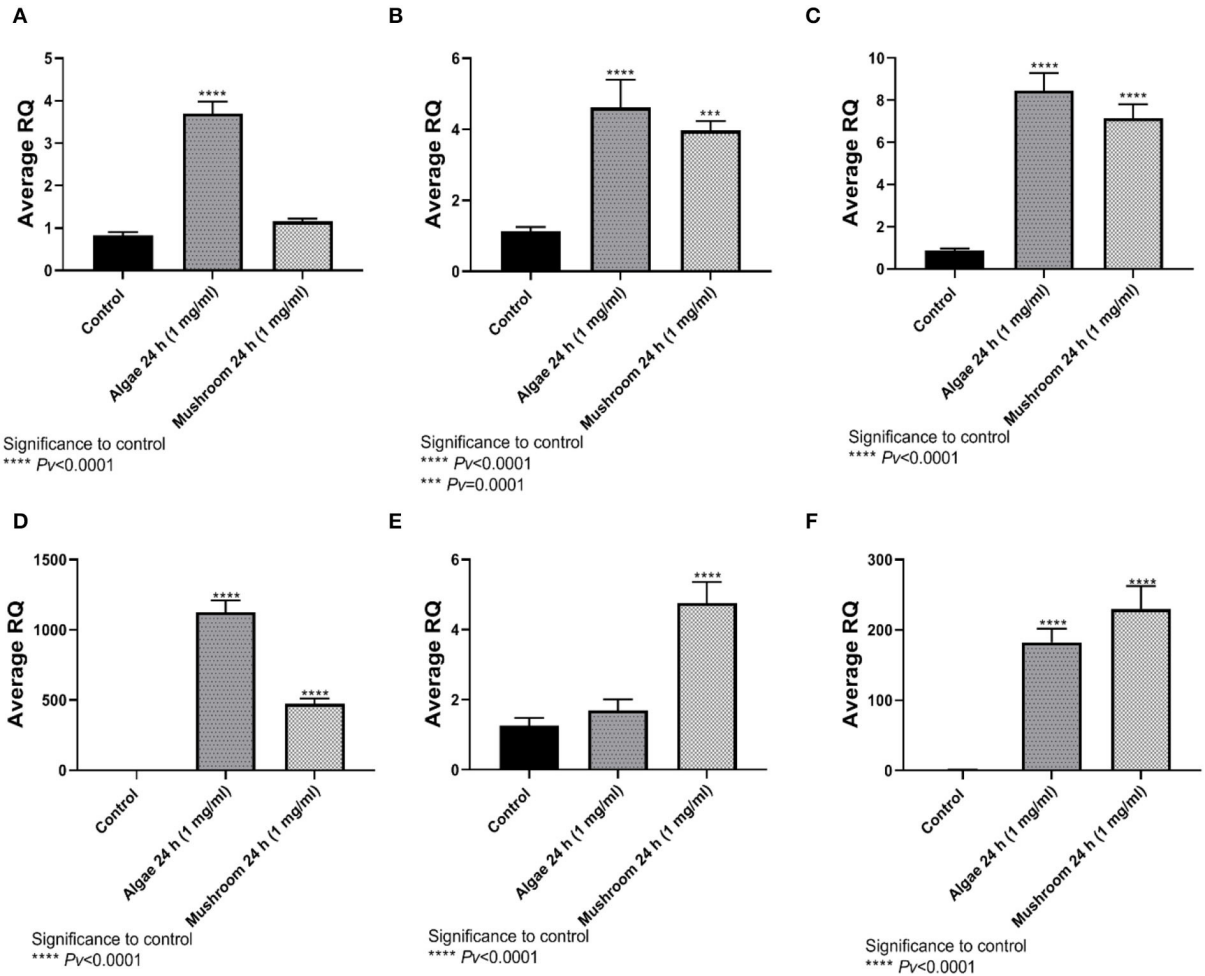
Gene expression after 24 h incubation with Algea or Mushroom glucans. **(A)** Tnfα, **(B)** IL4, **(C)** IL6, **(D)** IL8, **(E)** IL10, **(F)** iNOS_2_ compared to the control group using the relative fold gene expression (DDCT) (n = 5).

TNFα expression was significantly higher than control after exposure to algae beta-glucans, but no significant expression was measured when exposed to beta-glucans extracted from mushrooms (Figure 4A). The expression of IL4, IL6, and IL8 was significantly different from the control after 24 h of exposure to algae beta-glucans and mushrooms beta-glucans (Figures 4B–D). IL10 expression was significantly different from the control after exposure to mushrooms beta-glucans, but no significant expression was measured when exposed to algae beta-glucans (Figure 4E). iNOS, similarly to IL4, IL6, and IL8, expressed significantly from the control after 24 h of exposure to both algae and mushrooms beta-glucans (Figure 4F).

#### 4.2.2. Nitric oxide secreation

**Figure 5:**
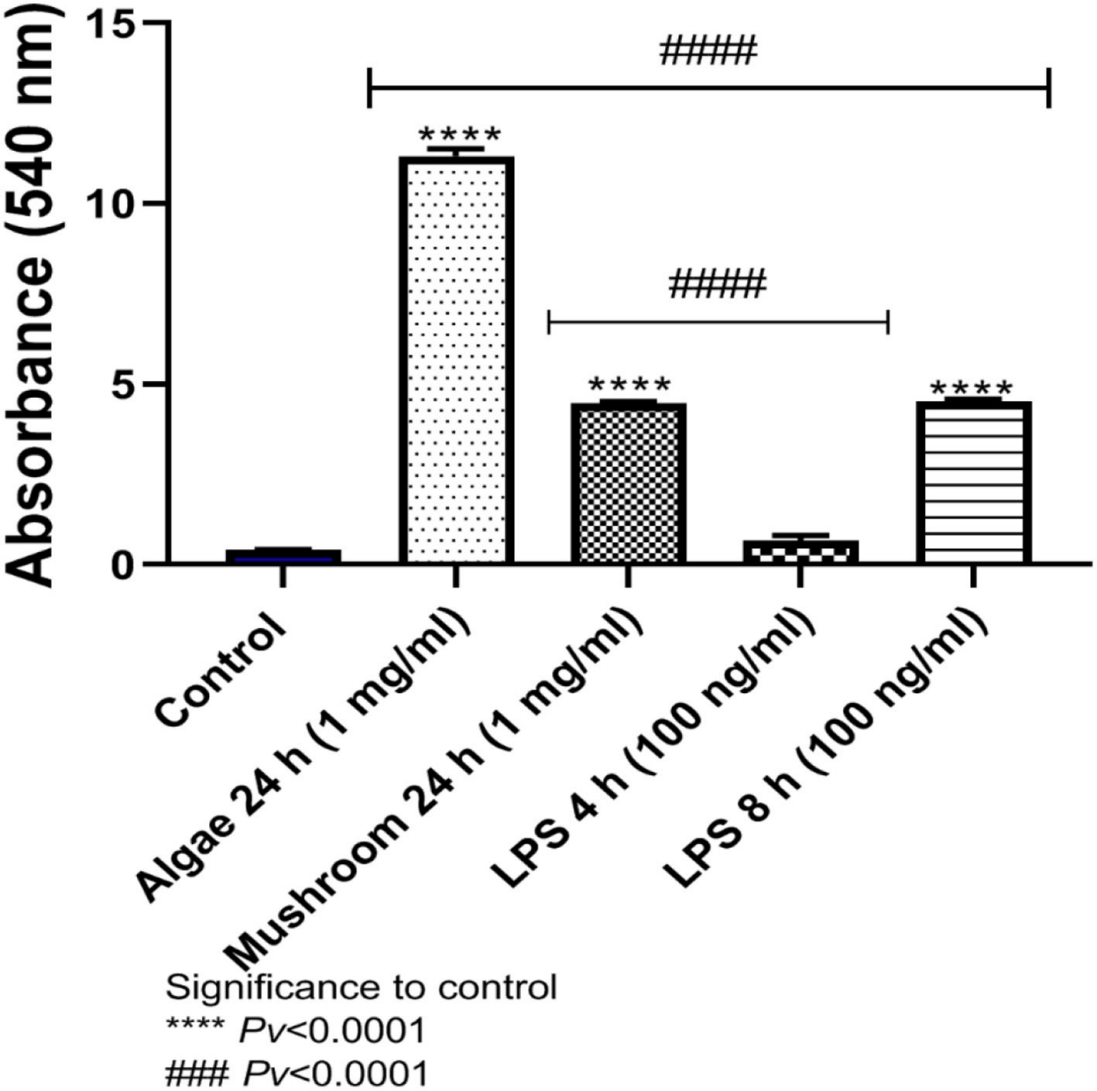
Release of NO to the medium after exposure to algae or mushrooms beta-glucans glucans for 24 h, or LPS. ****Pv < 0.0001 significance to control group. ^###^Pv < 0.0001 significance between beta-glucans to LPS for 4 and 8 h (n = 5).

The release of NO to the medium after exposure to either mushrooms or algae beta-glucans for 24 h, and 1 mg/ml beta-glucans, was significantly greater compared to the control group. Algae beta-glucans induced a release of more NO than the groups exposed to LPS for either 4 or 8 h. These results are consistent with our previous results related to NO gene expression. In contrast, the amount of NO released after exposure to mushroom beta-glucans was significantly different from the group exposed to 4 h of LPS but not the one exposed to 8 h of LPS (Figure 5).

#### 4.2.3. Phagocytosis

**Figure 6:**
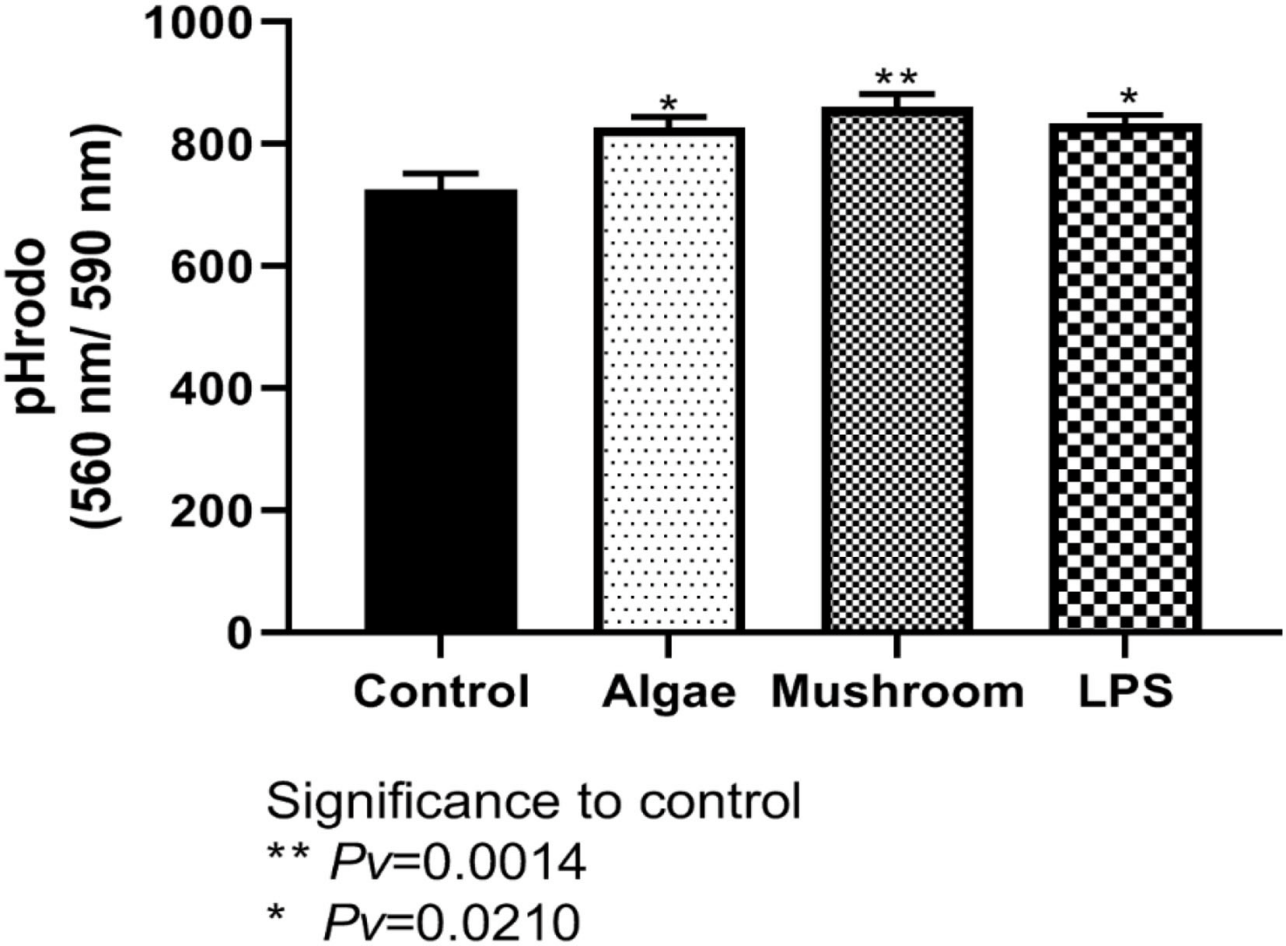
Measured phagocytosis activity after 60 min. HD11 cell line were incubated for 1 h with 1 mg/ml of either mushrooms or algae beta-glucans (n = 6), or 100 ng/ml of LPS (n = 3) and compared to control (n = 3).

To examine phagocytosis activity, HD11 cells were incubated for 1 h with 1 mg/ml of either mushroom or algae beta-glucans or 100 ng/ml of LPS. Phagocytosis was measured using pHrodo™ Red Zymosan BioParticles (“Zymosan,” Life Technologies, USA) after 60 min (Figure 6). Algae and mushroom beta-glucans increased phagocytosis activity significantly compared to control at both time points. LPS increased phagocytosis activity significantly from the control only after 60 min (Figure 6).

#### 4.2.4. Vili size

**Figure 7:**
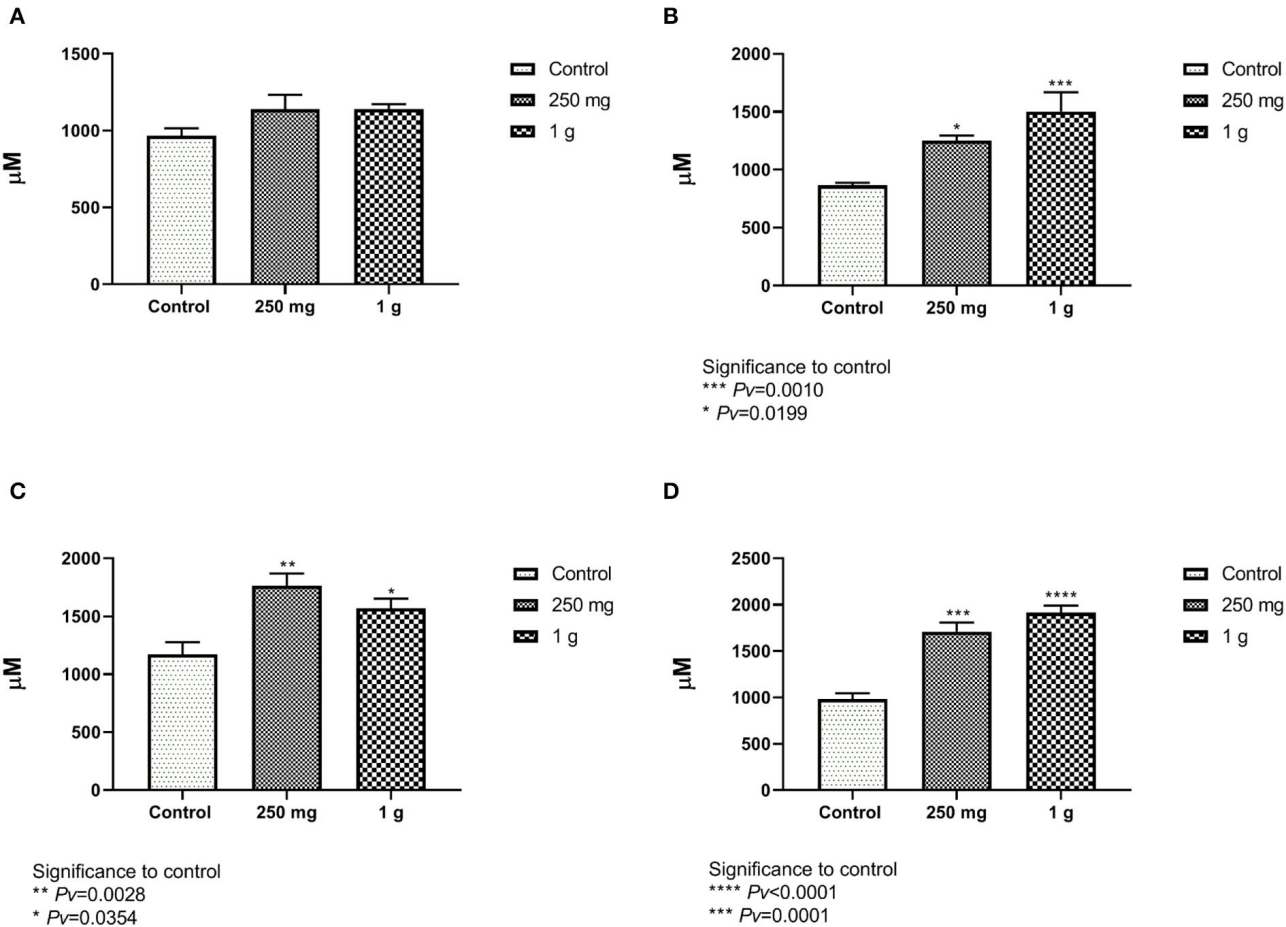
**(A, B)** The length of the villi from the ileum compared with the beta-glucans treatment groups to the control. **(A)** The females and figure. **(B)** The males (n = 5 per group). **(C, D)** The length of the villi from the jejunum compared with the beta-glucans treatment groups to the control. **(C)** Represent the females, and **(D)** for the males (n = 5 per group).

The increase of villi height directly affects the nutrient absorption capability in the intestine as it would increase the absorptive and surface area (24). The villi height was defined as the distance from the villus tip to the crypt junction. Measurements of the villi taken from the female ileum showed no differences between treatment with glucan #300 beta-glucans to control (Figure 7A, Supplementary Figure 4). The length of the male villi from the ileum was significantly longer in both treatment groups compared to the control (Figure 7B). Measurements of the female and male villi from the jejunum showed significant differences between treatment with glucan #300 beta-glucans to control (Figures 7C, D).

#### 4.2.5. Goblet cell count

**Figure 8:**
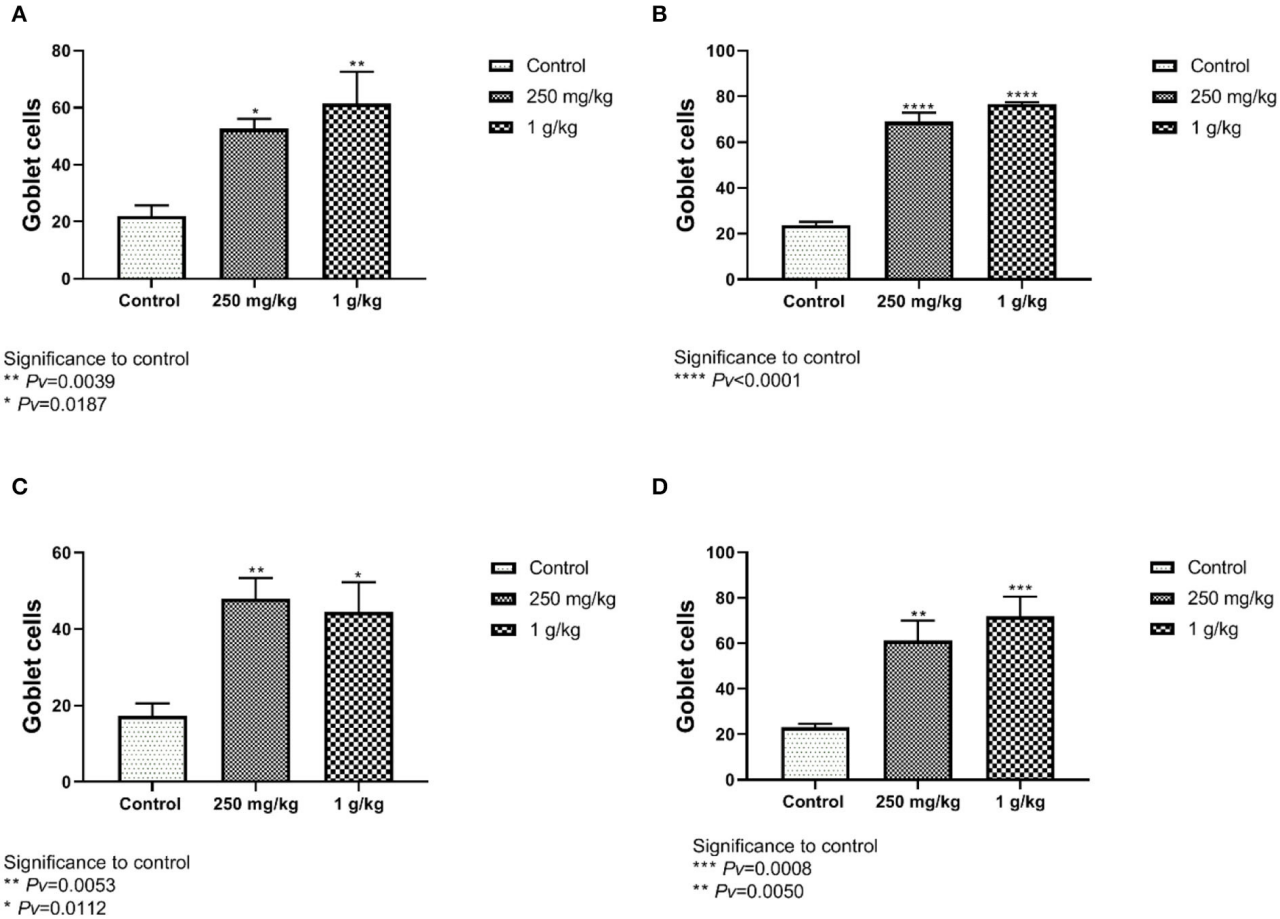
The number of goblet cells counted from the jejunum and ileum sections. Beta-glucans treatment groups compared to the control. **(A)** Females ileum, **(B)** male ileum, **(C)** female jejunum, **(D)** male jejunum (n = 5 per group). Pv is compared to control.

The number of goblet cells from the ileum and the jejunum of the females showed significant differences between both treatments, 1 g/kg and 250 mg/kg, with beta-glucans to control (Figures 8A, C, Supplementary Figure 5). These results were also evident in the male group (Figures 8B, D).

### 4.3. Effect of a combination of yeast and mushroon glucans

**Figure 9:**
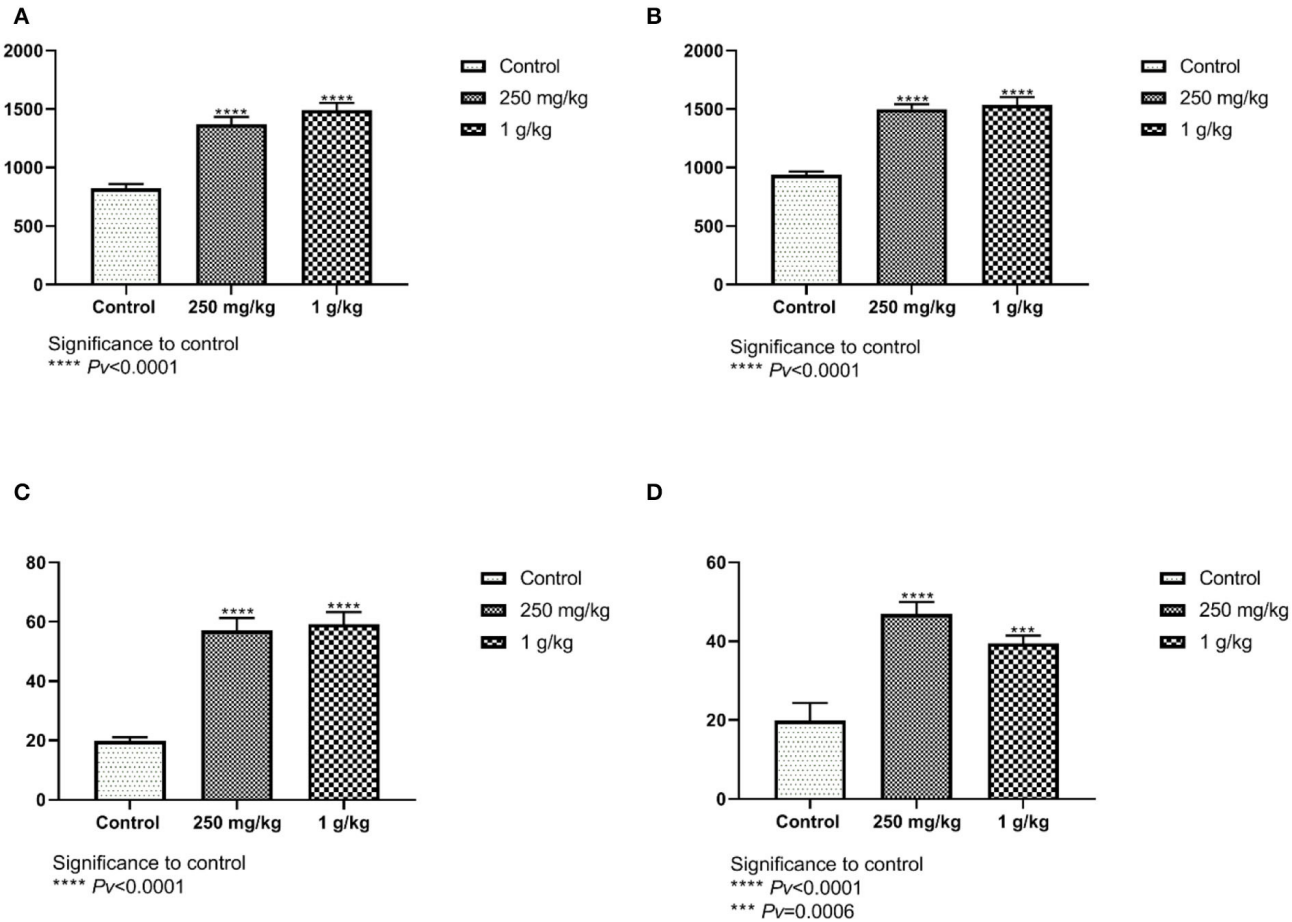
**(A, B)** The length of the villi from the jejunum compared with the beta-glucans treatment groups to the control. **(A)** Females, and **(B)** males (n = 10 per group). **(C, D)** The number of goblet cells in the jejunum. Beta-glucans treatment groups compared to the control. **(C)** Females, and **(D)** males (n = 10 per group). Pv are compared to control.

Measurements of the villi taken from the female jejunum showed significant differences between treatment with beta-glucans to control. The length of the female and male villi from the jejunum was significantly longer in both treatment groups compared to the control (Figures 9A, B). The number of goblet cells found in the jejunum of the females showed significant differences between both treatments, 1 g/kg and 250 mg/kg beta-glucans treatments as compared to control (Figure 9C). These results were also observed in the male group (Figure 9D). Similar results were obtained for ileum length and goblet cell number measurements (not shown).

## 5. Discussion

Bacterial infections in poultry are an essential concern for both animal health and productivity. The current treatment in poultry farming relies heavily on antibiotics to prevent disease outbreaks (2). It is known that over 60% of all antibiotics produced worldwide, find their use in animal production for both therapeutic and non-therapeutic purposes (25). The massive use of antibiotics led to an increased risk for pathogen resistance. The result has been the withdrawal of several antibiotics from the toolbox available to poultry producers (9). The potential risk of antibiotic resistance resulted in the ban of some antibiotics as feed additives by the European Union as of January 1, 2006. Unfortunately, this ban has led to a decline in animal health and more significant illness variability (26).

Beta-glucans are polymers of glucose that can be derived from the cell walls of yeast, bacteria, fungi, and cereals (27). Considerable variation exists in the structure of beta-glucans from these different sources, which ultimately results in differences in their physiological functions (28). The effects of beta-glucans as biologically active immunomodulators have been well noted in mammalian species; while the mammalian and avian immune systems are similar, they are different enough that it is difficult to extrapolate mammal research to poultry. Recent research shows that beta-glucans may play a role in replacing antibiotics and stimulating the immune system in the poultry industry (16).

Dietary supplementation of yeast cell wall beta-glucans induced a moderate weight gain in the spleen and the bursa of Fabricius (12). In contrast, Cox et al. (16) found no significant differences in total body weight and in body weight gain among the treatment groups with beta-glucans. These inconsistent results could be attributed to various reasons, such as differences in the source of the beta-glucan, the presence and type of challenge used, or both. As such, it emphasizes the importance of further research to pinpoint the optimal dosage of beta-glucan supplementation for consistently favorable results in poultry.

The results of the present study suggest that incorporating beta-glucans into the chickens' diet significantly improves gut health by priming intestinal macrophage function and thus affecting intestinal morphology and function, resulting in effective anti-inflammatory immunomodulators. Beta-glucan supplementation benefits the number of goblet cells and the villi height in both the jejunum and the ileum. Moreover, beta-glucans supplementation increased the phagocytic function of macrophages and enhanced the immune response by altering the cytokine profiles of chickens. As alluded to earlier, during ingestion beta-glucans reach the intestine and from their pass through the Peyer's patches into the GALT, and subsequently, they are transported around the body (9). In this way, beta glucans from food can directly stimulate macrophages and affect their secretion of immune-related molecules.

Chicken macrophages are the most potent antigen-presenting cells capable of resistance to exogenous pathogenic microorganisms (14). Dietary supplementation with beta-glucans to poultry has been demonstrated to stimulate phagocytosis, which eventually suppresses the invasion of the pathogen into organs (29). Additionally, beta-glucan supplementation increased the phagocytic function of macrophages as we show herein and as previously published (30).

Macrophages have been extensively studied in several mammalian species, but little attention has been given to the study of avian macrophages. Our *in vitro* experiment was conducted using macrophages originated from a transformed chicken macrophage cell line, HD-11. To our knowledge, no previous research was conducted using the HD-11 macrophage cell line to test the effect of beta-glucans. Other cell lines were assayed for the effect of beta-glucans, mainly chicken MQ-NCSU macrophage cells (30).

To understand the immunomodulatory effect of beta-glucans HD-11 chicken macrophage cell line was used. We measured the expression of 6 genes at different time points and concentrations. Significant expression from control was measured with all 6 genes (TNFα, IL4, IL6, IL8, IL10, and iNOS_2_). To stimulate the maximal response of secretion, we choose to use a concentration of 1 g/ml of beta-glucans for 24 h of incubation (Figures 1, 2). When comparing yeast beta-glucans to mushroom β-glucans expression of IL4, IL6, IL8, and iNOS were significantly higher than control after 24 h of exposure to both algae and mushrooms beta-glucans (Figure 4). IL10 expression was significantly different from the control after exposure to mushrooms beta-glucans, but no significant expression was measured when exposed to algae beta-glucans (Figure 4F). Zhang et al. (31) observed a quadratic increase in the cytokines levels of TNFα and IFN-γ in the blood serum of chicken after being fed with either 50 or 75 mg/kg/feed beta-glucans. Supplementation with beta-glucan for 7-days post hatch up-regulated IL4 expression in the duodenum, jejunum, and ileum. By day 14, IL4 expression was up-regulated in the duodenum and ileum (16). Queiroz et al. (32) reported that a concentration of 50 mg/kg/fed beta-glucan from mushrooms increased the level of IL10 concomitant with a reduction of IFN-γ, in poultry chickens.

In contrast, when compared with un-supplemented controls, broiler chickens who received beta-glucan from yeast were shown to down-regulate IL8. The down-regulation of the IL8 gene with beta-glucan supplementation suggests that the beta-glucan functions as an anti-inflammatory immunomodulator (16). Qureshi et al. (30) reported that no detectable IL6 bioactivity was observed in macrophage culture supernatants with or without beta-glucan exposure. This result may be explained since their experiment was conducted using the MQ-NCSU macrophage cell line, which is derived from the spleen and may not express IL6, in contrast to HD11, who is derived from chicken bone marrow and does express IL6. We suggest that care should be undertaken in regard to the different data published in regards to the various effects caused by beta-glucans. Since many products may be called beta-glucans, the composition and analytical conformation is likely to differ from source to source, which may influence the vast variety of responses observed.

NO is an important molecule in the anti-inflammatory and antimicrobial effects of macrophages and is a chemical indicator of inflammation and inflammatory diseases. iNOS is induced in inflamed tissues and generates relatively large amounts of NO (33).

When exposed to antigens or chemotactic agents, macrophages will begin to express iNOS. This enzyme leads to the production of NO, which will subsequently react with superoxide anions to generate toxic derivatives, allowing macrophages to proficiently kill several types of pathogens (34). The upregulation in iNOS implies an enhanced capability of macrophages to kill invading pathogens, allowing the host to eliminate infectious threats more efficiently (16). Beta-glucan exposure increased NO release into the supernatant fraction of broiler macrophage culture, suggesting that beta-glucans may induce nitric oxide synthase activity similar to other known macrophage activators such as LPS (35). The release of NO to the medium after exposure to 1 mg/ml of either mushrooms or algae beta-glucans for 24 h or 100 ng/ml of LPS for 4 and 8 h was measured. The results showed a significantly greater NO release for all treatment groups than the amount released from the untreated control group. Exposure to algae beta-glucans induced the released of more NO than the groups exposed to LPS for either 4 or 8 h (Figure 3). These results were consistent when comparing algae and mushroom beta-glucan NO release (Figure 5) and the significant expression of the nitric oxide synthase (iNOS) gene after exposure to beta-glucans for 24 h (Figures 2D, 4F).

Macrophages are one of the first responder innate immune cells upon a new infection, as seen in infection models that show the infection causes a quick increase in the number of macrophages (36). They can phagocytize bacteria and subsequently produce multifunctional compounds, including reactive oxygen species (ROS), nitric oxide (NO), and cytokines, to kill the infectious microorganisms and signal to other immune cells to establish an appropriate response to the infection (37). Moreover, activated macrophages can produce NO, which plays an important role in the host defense against microbial infection, and act as effector molecules to kill invading pathogens (38). The release of NO to the medium after exposure to beta-glucans was observed in our research and can be explained by the increase of phagocytosis activity. Many studies on phagocytic activity have been described for mammalian macrophages, but the available data for chicken macrophages is limited.

Besides the intracellular killing of pathogens, professional phagocytes play an important role in modulating the immune response by expressing cytokines and chemokines. For example, induced pro-inflammatory cytokines IL8 as well as inflammatory cytokine IL6 (38), which was also observed in our research. Phagocytosis is a complex process involving a diverse set of receptors that can stimulate phagocytosis. Cells were incubated for 1 h with 1 mg/ml of either mushroom or algae beta-glucans or 100 ng/ml of LPS. Algae and mushroom beta-glucans increased phagocytosis activity significantly from the control (Figure 6). From the following data it can be concluded that β-glucans from both algae and mushroom can elicit a proinflammatory response in HD-11 avian macrophages. This mild proinflammatory response can prime macrophages to be active for subsequent putative infections.

Longer intestinal villi indicate an improved ability to absorb nutrients in the intestine and may account for increased body weight (39). The crypts of the villus contain several specialized cells, including absorptive cells, goblet cells, and regenerative cells, that are responsible for the production of mucus and the replacement of old cells (15). Assessment of intestinal villi morphology is important since the small intestine is the primary site for nutrient assimilation and is therefore sensitive to diet changes (40). Measurements of the villi taken from the female ileum showed no differences between treatment with beta-glucans to control (Figure 7A). The length of the male villi from the ileum was significantly longer compared to the control (Figure 7B). Measurements of the female and male villi from the jejunum showed significant differences between treatment groups to control (Figures 7C, D, 9A, B). Correlating with our findings, several studies have also observed that dietary yeast beta-glucan administration to chickens resulted in higher villi height in the jejunum compared to chickens fed with no beta-glucan (40–42). Confirming our studies Solis de Los Santos et al. (15) found that the villi height of the ileum, the surface area, the lamina propria thickness, the crypt depth and the goblet cell density were enhanced due to the consumption of yeast extract beta-glucans as a dietary supplement.

The intestinal lining provides the innate defense barrier against most intestinal pathogens. Goblet cells in the intestinal mucosa secrete mucus which provides the first line of defense against intestinal injury (9). The mucus layer protects the intestinal surface from the invasion of enteric bacteria, bacterial and environmental toxins, and some dietary components that may damage the mucosa (15, 42, 43). When the mucus layer is disturbed, the adhesion of microbes to the intestinal epithelial surface may increase epithelial permeability and reduce the absorption of nutrients (44, 45). We measured herein whether the number of goblet cells was affected by the beta-glucan treatment. Goblet cell number from the ileum and the jejunum of the males and females showed significant differences between treatments with beta-glucans to control (Figures 8A–D, 9C, D). Dietary beta-glucan administration significantly increased the number of goblet cells in the jejunum and ileum. Goblet cells are a type of intestinal mucosal epithelial cells whose primary function is to synthesize and secrete mucus. Mucin production and secretion are important in maintaining the mucus barrier. A wide range of factors, including feeding, microbes, microbial products, toxins, and cytokines, has been shown to regulate these processes, thus affecting the mucus barrier (44–46). Correlating with our findings, previous studies also found that supplementation with beta-glucan significantly increased the number of goblet cells in the jejunum of chickens (15, 41). This suggests that beta-glucan supplementation of poultry feed plays a significant role in improving their gut health during a bacterial challenge.

## 6. Conclusions

Cumulatively, our results indicate that beta-glucans can influence cytokine expression profiles indicating that avian macrophages may express beta-glucans receptors. We conclude that algae and mushrooms beta-glucans modulate differently the expression of cytokines-associated with immune response suggesting that the use of the two beta-glucans types may result in a broader immune response.

Our findings are important since a healthier gut indicates better digestion and absorption of nutrients, and therefore a healthy functioning immune system. Similarly extrapolating our findings to human set-ups, the rationale of using adjuvants in vaccine formulations in order to improve the efficacy of adaptive immunity is widely accepted, however little attention has been given to the direct effects of adjuvants on innate immunity and early protection against infection (15, 44). As such, beta-glucans may enhance resistance to acute or chronic human infections and reduce the wide use of antibiotics.

**Institutional Review Board Statement**: “The study was conducted in accordance with the Dec-laration of Helsinki, and approved by the ethics committee of the Hebrew University of Jerusalem. The experiment was conducted at the poultry unit and research laboratories at the Faculty of Agriculture, Food and Environmental Sciences, Israel. All animal experiments were done under the care and supervision of Prof. Israel Rozenboim. Protocol code AG-20-16348-4, NIH approval number OPRR-A01-5011, date of approval, 28/10/2020.

## Data availability statement

The raw data supporting the conclusions of this article will be made available by the authors, without undue reservation.

## Ethics statement

The animal study was reviewed and approved by the study was conducted in accordance with the Dec-laration of Helsinki, and approved by the Ethics Committee of the Hebrew University of Jerusalem. The experiment was conducted at the poultry unit and research laboratories at the Faculty of Agriculture, Food and Environmental Sciences, Israel. All animal experiments were done under the care and supervision of Prof. Israel Rozenboim. Protocol code AG-20-16348-4, NIH approval number OPRR-A01-5011, date of approval, 28/10/2020.

## Author contributions

Conceptualization: BS, IR, and VV. Methodology: HB-D, OG, and NC. Writing-original draft preparation: OG, HB-D, and BS. Writing-review and editing, supervision, and funding acquisition: BS and IR. All authors have read and agreed to the published version of the manuscript.
