# Supplementary material for: Beta-glucans induce cellular immune training and changes in intestinal morphology in poultry
Source: Front Vet Sci. 2023 Jan 9;9:1092812. doi: 10.3389/fvets.2022.1092812 (PMC9868956; doi:10.3389/fvets.2022.1092812)
Supplement: Supplementary file 1 [file Data_Sheet_1.PDF]

Supplemental figures:

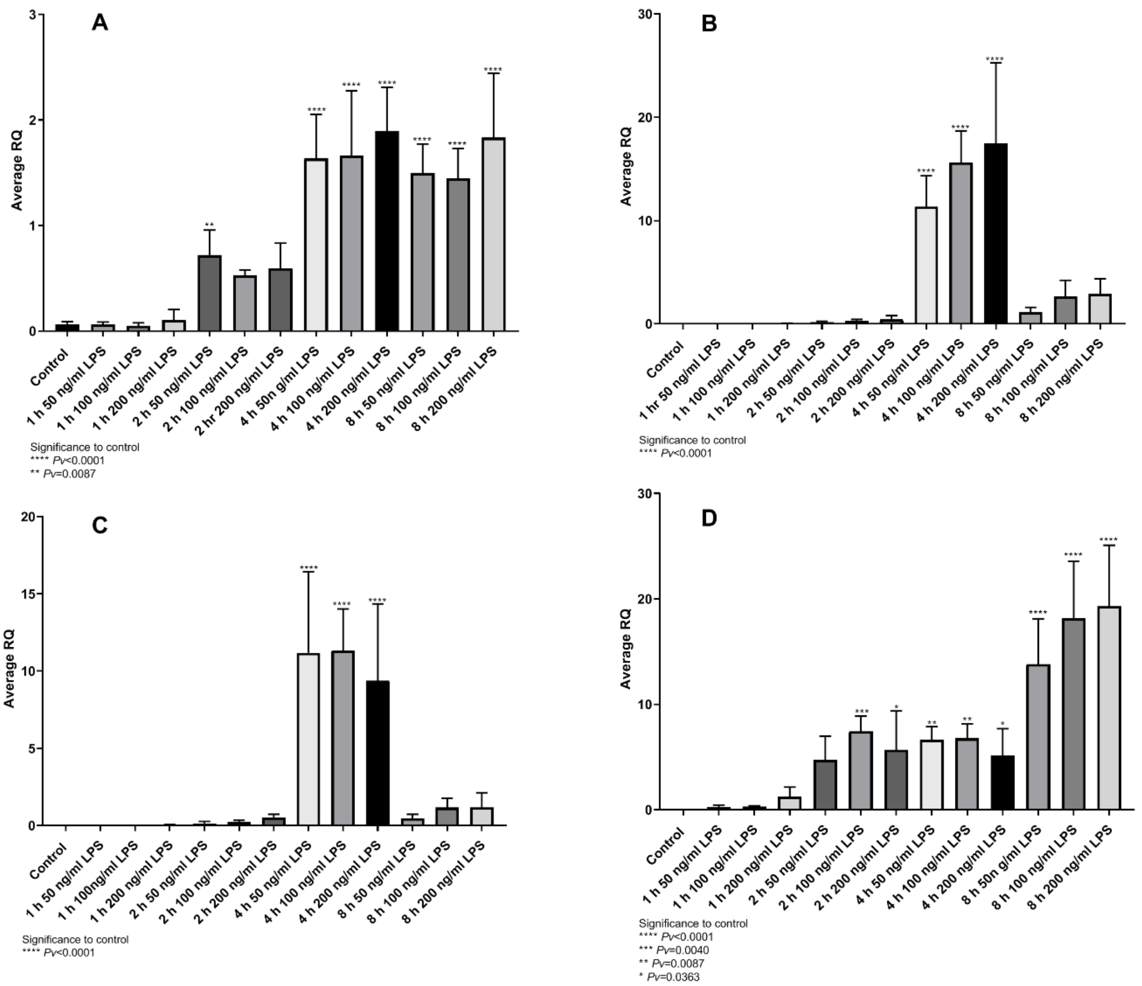

Supplemental figure 1: Average relative expression of genes to selected cytokines at 4 different time points and 3 different LPS concentrations. The expression increased significantly from the control levels, after 4 hours and all LPS concentrations. A. TNFα, B. IL4, C. IL6 and D. IL8. *P*v compared to control.

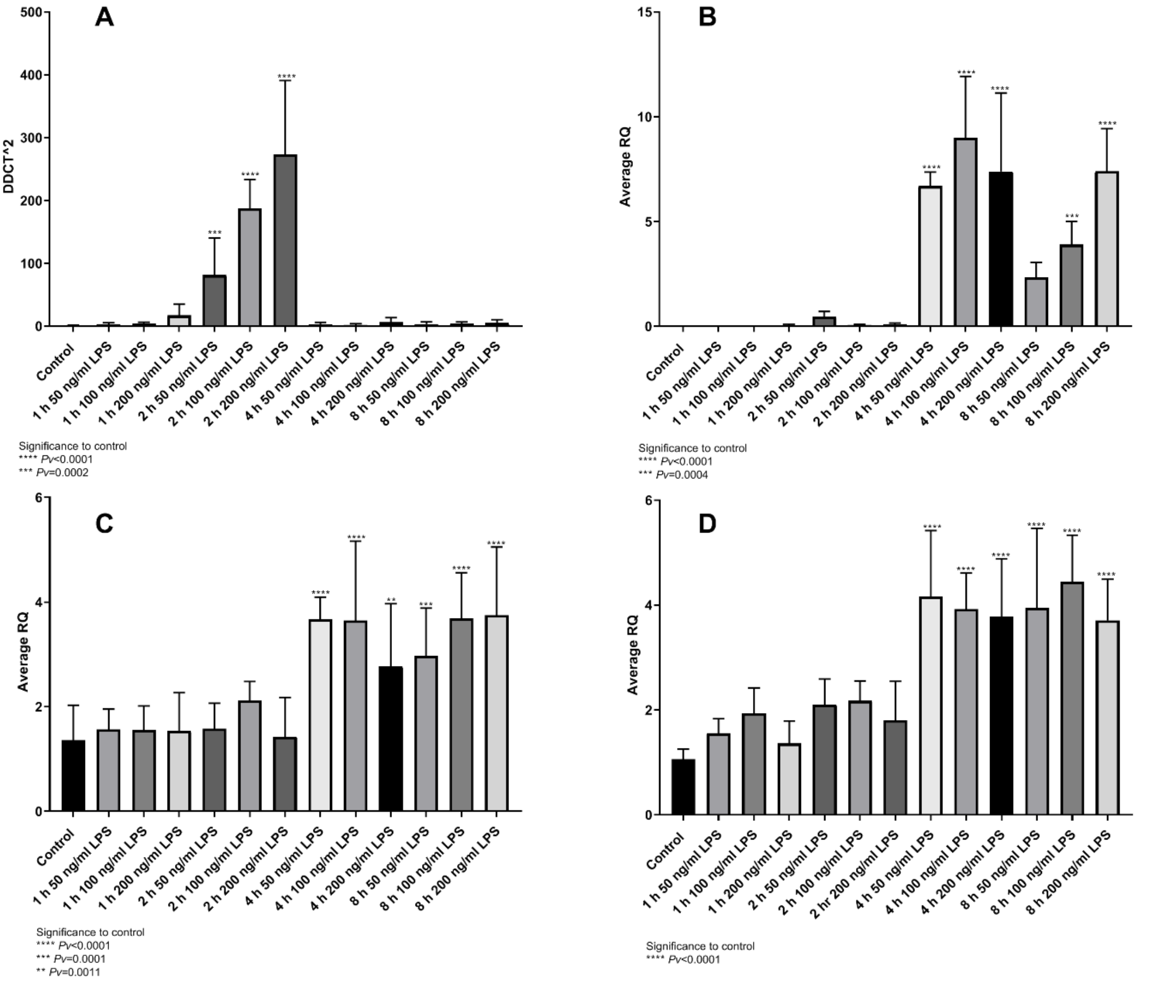

Supplemental figure 2: Average relative expression of genes to selected cytokines at 4 different time points and 3 different LPS concentrations. A. IL10, B. INF-γ, C. Cytochrome-C, D. BAX. *P*v compared to control.

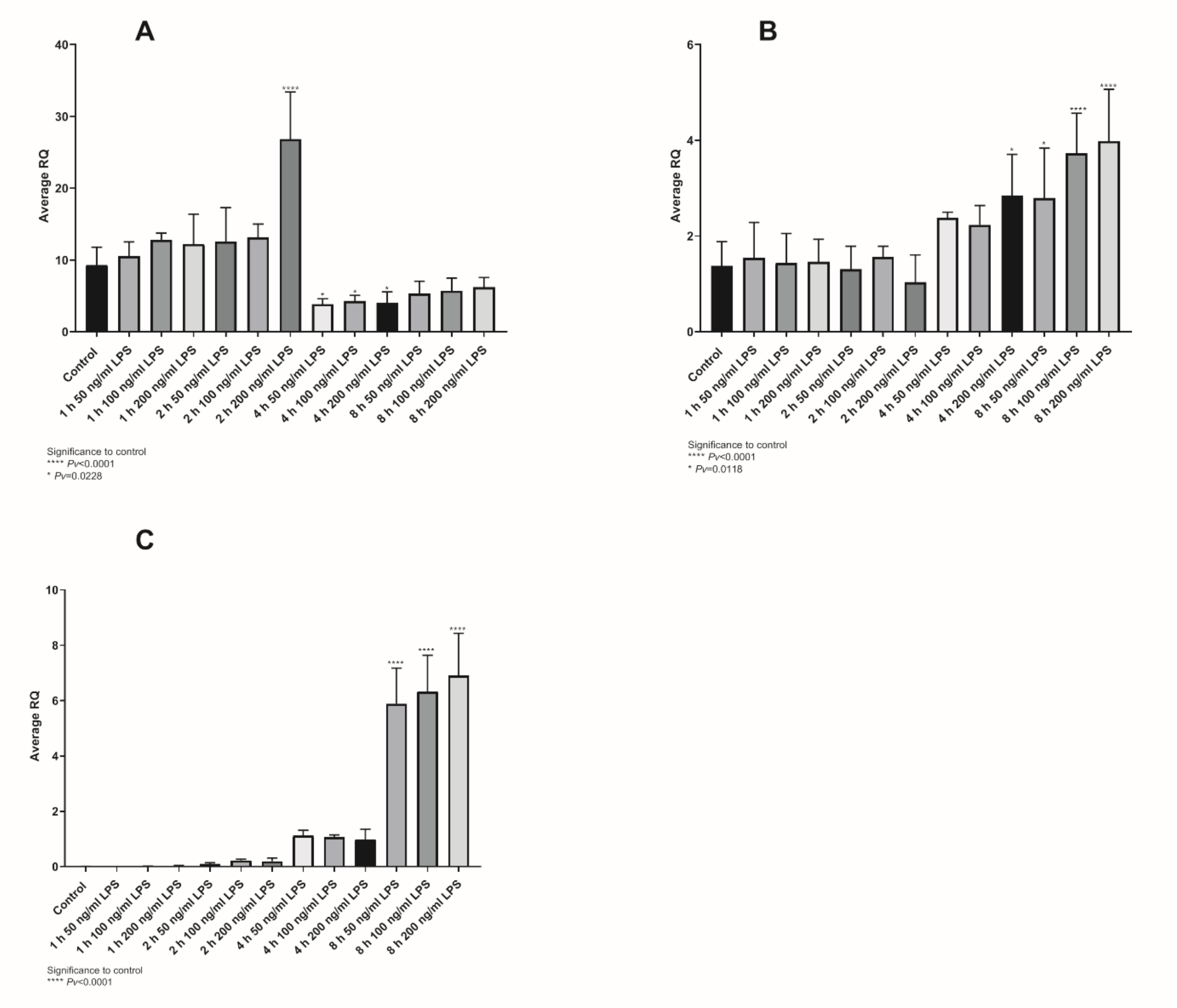

Supplemental figure 3: Average relative expression of 4 different genes at different time points and at 3 different concentrations. A. BCL2, B. Caspase 9, C. iNOS2. *P*v compared to control.

A **B**

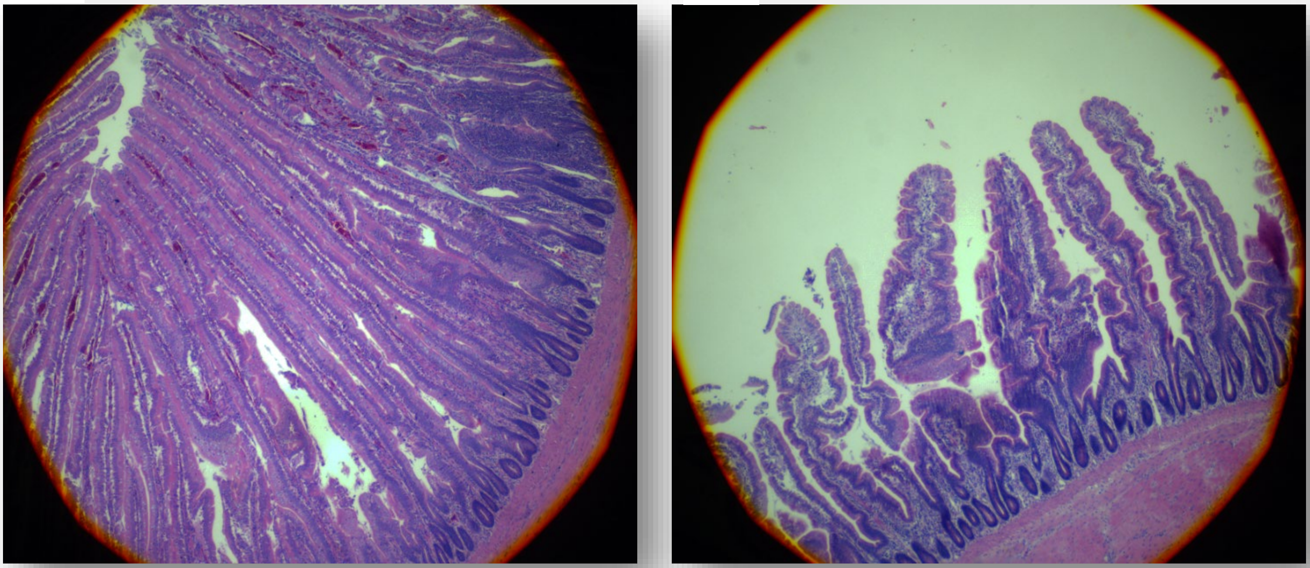

Supplemental figure 4: Representative images; A: Male, villi from the jejunum, treatment 1 g/kg glucans. B: Male, villi from the jejunum, control.

Microscope images obtained at x 10 magnification.

A B

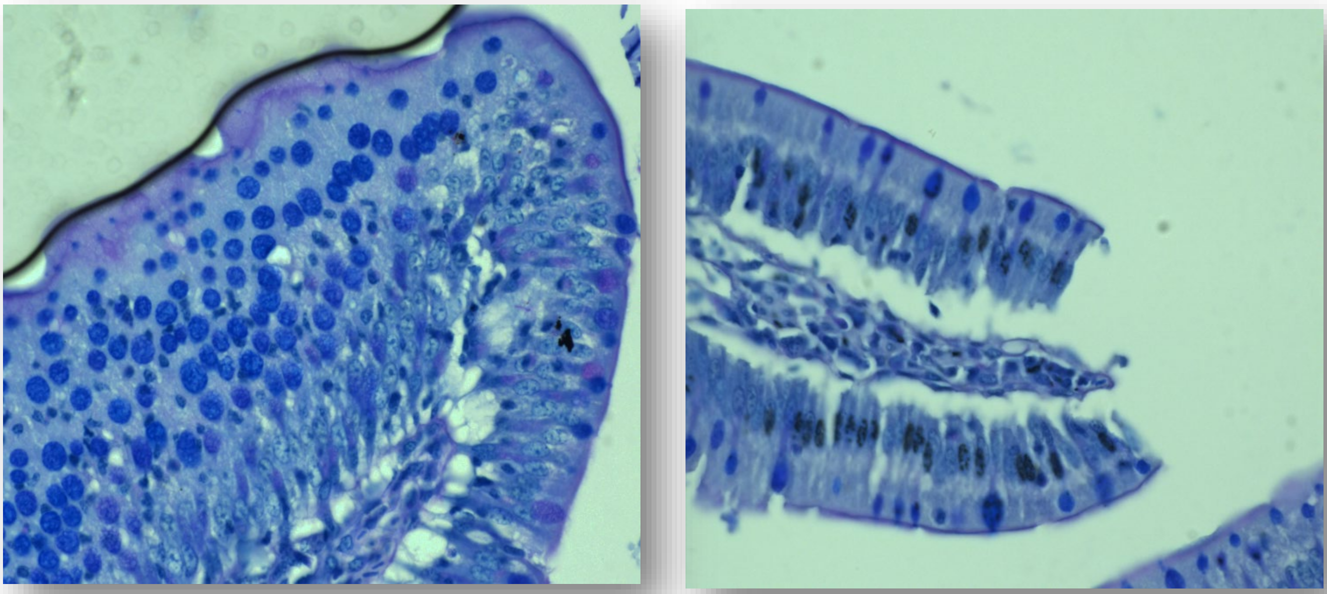

Supplemental figure 5: Representative images; A Female, goblet cells from the ileum, treatment 1 g/kg glucans. B: Female, goblet cells from the ileum, control. Microscopic images obtained at x 40 magnification.
